# Imprinting disorders as a window to understand pediatric feeding disorders

**DOI:** 10.1186/s13023-025-03789-y

**Published:** 2025-05-24

**Authors:** Juliette Salles, Thomas Gorse, Grégoire Benvegnu, Jean-Philippe Raynaud, Maithé Tauber

**Affiliations:** 1 Service de psychiatrie d’urgence de crise et de liaison, CHU de Toulouse, Toulouse, France; 2 Infinity (Toulouse Institute for Infectious and Inflammatory Diseases), INSERM UMR1291, CNRS UMR5051, Université Toulouse III, Toulouse, France; 3 Service Universitaire de Psychiatrie de l’Enfant et de l’Adolescent (SUPEA), CHU de Toulouse, Toulouse, France; 4 Centre de compétences Maladies Rares à Expression Psychiatrique, Service Universitaire de Psychiatrie de l’Enfant et de l’Adolescent, CHU de Toulouse, Toulouse, France; 5 Centre de référence Maladies Rares (PRADORT, Syndrome de Prader‑Willi et autres formes rares d’obésité avec troubles du comportement alimentaire), Service d’Endocrinologie, Obésités, Maladies Osseuses, Génétique et Gynécologie Médicale, ENDO-ERN (European Reference Network on Rare Endocrine Conditions), Hôpital des Enfants, CHU de Toulouse, Toulouse, France; 6 CERPOP (Centre d’Epidémiologie et de Recherche en santé des POPulations), UMR1295, INSERM - Université Toulouse III Paul Sabatier, Toulouse, France; 7 Centre de Référence du Syndrome de Prader-Willi et Syndromes avec Troubles du Comportement Alimentaire, Hôpital des Enfants, Unité D’endocrinologie, Obésités, Maladies Osseuses, Génétique et Gynécologie Médicale, CHU de Toulouse, Toulouse, France

**Keywords:** Pediatric feeding disorders, Oxytocin, Hypothalamus, Imprinting disorders

## Abstract

Imprinting disorders are a group of rare congenital disorders characterized by common clinical features that affect growth, development, metabolism, and shared molecular abnormalities [1]. Patients with these disorders exhibit feeding difficulties and changes in social skills. Pediatric feeding disorders affect approximately 25% of children in the general population but have been difficult to understand and manage globally; indeed, they have traditionally been approached from different professional disciplines, each advocating its own unique method. An interdisciplinary consensus group recently introduced a more integrative definition of pediatric feeding disorders. From this new approach, we hypothesized that the imprinted genes may play an important role in the relationship between feeding and social development. In addition, we hypothesize in this letter that research on imprinting disorders may contribute to a better understanding of pediatric feeding disorders.

## Imprinting disorders and imprinted genes

Imprinting disorders represent a group of rare congenital conditions characterized by shared clinical features affecting growth, development, metabolism, and common molecular disturbances. These disorders are related to the imprinting system, which is thought to have arisen in response to unique selective pressures associated with pregnancy. Recent research suggests the existence of an imprinted gene-dependent ‘loop,’ wherein imprinted genes can influence specific maternal behaviors (e.g., maternal licking and grooming). These behaviors, in turn, affect the expression of genes that are particularly sensitive to epigenetic changes in their offspring, potentially involving imprinted genes themselves [2]. In humans, it is suggested that brain processes and behaviors linked to higher-level social communication, including speech, reading, and language, may be susceptible to parent-of-origin effects dependent on imprinted genes [3]. Genomic imprinting, an epigenetic process, results in parent-of-origin-specific gene expression in mammals. This phenomenon reflects a difference in interests between the maternal and paternal genomes. Maternal genes aim to conserve resources over multiple offspring, as females are related to all their offspring. In contrast, the opportunistic paternal genome, not necessarily related to other offspring of the mother, seeks to extract maximum maternal resources. Consequently, paternally expressed genes tend to promote growth, while maternally expressed genes restrict it [4, 5].

Studies in mice with manipulations of individual imprinted genes have unveiled diverse roles, including those related to maternal care, feeding, and social behavior [6, 7], indeed studies have shown that imprinted genes expressed in the offspring can influence maternal behavior by improving the quality of maternal care [8]. Data, also suggest that imprinted genes exert an important role on behavioral functions through influencing oxytocin (OXT) and vasopressin neuron development and/or survival [9]. OXT is a neuropeptide significantly implicated in social attachment and parent-infant interactions [10]. Various imprinted genes participate in neural differentiation and axonal outgrowth and the hypothalamus is notable for its high concentration of imprinted genes [11]. Interestingly, OXT is product by neurons of the paraventricular (PVH) and supraoptic (SON) hypothalamic nuclei. In addition to its role in the OXT system, the hypothalamus plays a role in feeding through other regions, including the ventromedial hypothalamus (VMH), lateral hypothalamus (LH), and arcuate nucleus (ARC), and arcuate nucleus (ARC), which secrete various neuropeptides such as neuropeptide Y (NPY) and agouti-related peptide (AgRP), which stimulate appetite (orexigenic), or proopiomelanocortin (POMC), which suppresses appetite (anorexigenic). Furthermore, the hypothalamus receives and integrates signals from the body, relaying information about energy status. The hypothalamus therefore plays a central role in social interaction and feeding.

Infants and children diagnosed with imprinting neurodevelopmental syndromes exhibit a range of feeding disorders, including anorexia and sucking deficits. These conditions are often associated with impaired endocrine and neurological development, as well as alterations in social skills. These alterations may be linked to varying degrees of hypothalamic dysfunction [12]. Although imprinted disorders are not universally associated with feeding disorders or uniformly exhibited feeding difficulties, the following description will focus on imprinting disorders that present with impaired social interactions and early feeding difficulties.

Prader-Willi syndrome (PWS) (OMIM #176270) is a rare genetic imprinting disorder occurring in approximately 1 in 20,000 births. This condition results from the loss of expression of the inherited paternal allele within the chromosomal 15q11-13 region. In the neonatal phase, individuals with PWS typically exhibit severe hypotonia (muscle weakness) and difficulties with suckling. They often display limited interest in feeding and fail to thrive, necessitating nasogastric tube feeding in about 80% of affected patients. These early symptoms serve as a strong indication for genetic testing. Early feeding issues in individuals with PWS have been extensively documented, and various nutritional phases have been defined. The initial phase, known as phase 1a, encompasses the first nine months of life and is characterized as follows: “Weak, uncoordinated suck. Usually, cannot breastfeed. Needs assistance with feeding either through feeding tubes (nasal/oral gastric tube, gastrostomy tube) or orally with special, widened nipples. Many would die without assisted feeding [13]. Additionally, individuals with PWS experience challenges in social interactions, exhibit inappropriate social inferences even when possessing comparable intellectual abilities, and struggle with theory of mind, which involves understanding the mental states of others [14]. They also display ritualistic behaviors akin to those found in autism and often exhibit intense temper outbursts. It is now acknowledged that the PWS phenotype arises from a hypothalamic dysfunction [15]. Our team especially demonstrated that intranasal OXT treatment administered to infants provided a positive effect on feeding and social skills, and administration to adults resulted in increased trust in others, decreased sadness tendencies and less disruptive behaviors [16, 17].

Schaaf-Yang syndrome (SYS) (OMIM # 615547) is a recently identified, uncommon imprinting disorder stemming from a mutation in the paternal *MAGEL2* gene located within the chromosomal PWS region. Its exact prevalence is not yet known. While early diagnosis can occur at birth or during the first months of life, it is often delayed until childhood or adolescence because the clinical features overlap with those of PWS. This syndrome is marked by a notably high occurrence of autism spectrum disorders (ASD), with 89% of SYS patients receiving an ASD diagnosis. Furthermore, research in Magel2 KO mouse models has revealed significant changes in the initiation of the suckling reflex. This alteration is observed in mouse models with reduced OXT release at birth, such as the Magel2-KO mouse, or in wild-type pups when treated with an OXT antagonist before their first suckling. Notably, in the case of Magel2-KO pups, this effect can be reversed through the administration of OXT within the first five hours [18].

Angelman syndrome (AS) (OMIM # 105830) is a neurogenetic disorder characterized by mental retardation, movement or balance disorder, typical abnormal behaviors, and severe limitations in speech and language. It is caused by the loss of expression of the UBE3A gene, which is located contiguously to the Prader-Willi Syndrome chromosomal region (15q11.2-q13). Increased appetite and behavioral orientation to food affect approximately one-third of patients [19]. Children who suffer from AS due to paternal uniparental disomy (pUPD) exhibited feeding problems, including a significant overeating behavior, and developed regular obesity after 2–3 years of age [20]. Considering social interaction, despite a tendency toward social gregariousness and positive interpersonal bias, patients with AS often encounter problems in everyday interaction because of poor detection and respect of emotional and social signals [21].

Silver-Russell syndrome (SRS) (OMIM #180860), another infrequent imprinting disorder, results from a microdeletion in the chromosomal 11p15.5 region, maternal uniparental disomy (mUPD) of chromosome 7, mutations in the maternally imprinted *IGF-2* gene, or other rare molecular abnormalities. SRS is characterized by severe growth retardation at birth (small for gestational age), distinct craniofacial features, body asymmetry, and feeding difficulties. Feeding issues arise from a reduced appetite and complications related to oral motor function, including food aversion or aspiration. In severe cases, gastrostomy or jejunostomy tube feeding may be necessary. The syndrome is also associated with social impairments and behaviors indicative of ASD. Patient with SRS are predicted to have twice the normal level of CDKN1C. A mouse model with elevated expression of Cdkn1c shows hypothalamic dysfunction with alterations in a number of neurons in this region [22].

Temple syndrome (TS14) (OMIM # 616222) is a rare imprinting disorder involving genes within the chromosome 14q32 region, within this region, the mutated DLK1 gene is highly expressed in the hypothalamus [23]. DLK1 has been implicated in postnatal development of hypothalamic functions, particularly those regulated by the arginine-vasopressin and OXT systems [24]. TS14 is characterized by neonatal hypotonia coupled with feeding difficulties, as well as growth and weight retardation (small for gestational age). Affected individuals may exhibit mild facial dysmorphism, including a prominent forehead, a short nose with a flat nasal root and wide tip, downturned mouth corners, a highly arched palate, and micrognathia. Approximately 43% of affected infants experience failure to thrive, with half of them requiring nasogastric tube feeding. While autism spectrum disorders are rarely reported, some individuals may face neurological and emotional challenges.

Beckwith-Wiedeman syndrome (BWS) (OMIM # 130650) is related to the mutation or deletion of imprinted genes within the chromosome 11p15.5 region. The clinical presentation is highly variable; some cases lack the hallmark features of exomphalos, macroglossia, and gigantism. BWS patients can show early-onset obesity, although BWS is often an underestimated cause of syndromic obesity [25]. In a cohort of adults with BWS, the final height was up to + 2 SDS in 44% of patients. Furthermore, 57.7% of cases demonstrated height above their genetic target [26]. Furthermore, children diagnosed with BWS exhibit emotional and behavioral difficulties, including increased anxiety, low self-esteem, social withdrawal and a tendency to control externalizing reactions [27].

## Pediatric feeding disorders new perspectives and future challenges

Pediatric feeding disorders (PFDs) are a concern for approximately 25% of children within the general population. In the past, these disorders were categorized using a distinction between organic and nonorganic factors, and the diagnosis of feeding disorders was traditionally approached from various professional disciplines, each advocating its own unique method. However, in 2019, an interdisciplinary consensus group introduced a more comprehensive definition of PFDs. They now define PFDs as impaired oral intake that deviates from age-appropriate norms and is accompanied by issues related to medical, nutritional, feeding skill, and/or psychosocial functioning [28]. They also suggest that developmental factors resulting in delays of motor skills, language, socialization, and cognition can contribute to the development of PFDs. Following this approach that consider feeding as a complex behavior finely tuned by genetics, brain functioning, and hormones, it seemed therefore appropriate to envisage a comprehensive model wherein environmental factors such as parent-infant interactions and individual factors including genetic, endocrinological, metabolic, gastrological, and psychiatric parameters are involved.

## Could the promotion of research on imprinting disorders be a pathway to the understanding of pediatric feeding disorders?

Therefore, we suggest that the comprehensive model for FEDs should further investigate the relationship between environmental factors (including social interactions) and feeding impairment. In addition, we postulate that delineating the role of imprinted genes known to be involved in hypothalamic function, including social and feeding function, may help to understand this relationship. Consequently, studies of imprinting disorders may be valuable in developing new knowledge in this area. This new perspective has the potential to improve our understanding of the clinical course of imprinting disorders and FEDs and may open avenues for identifying new targets for treatment and/or considering early OXT treatment in the postnatal period under certain circumstances.

## Data Availability

Not applicable.

## Abbreviations

ASD: Autism spectrum disorders
OXT: Oxytocin
PFD: Pediatric feeding disorders
PWS: Prader Willi Syndrome
SRS: Silver-Russell syndrome
SYS: Schaaf-Yang syndrome
TS14: Temple syndrome
